# Reduced hydrogen sulfide production contributes to adrenal insufficiency induced by hypoxia via modulation of NLRP3 inflammasome activation

**DOI:** 10.1080/13510002.2022.2163354

**Published:** 2023-01-20

**Authors:** Ningning Zhang, Zhan Zhou, Yan Huang, Gang Wang, Zhengshan Tang, Jianqiang Lu, Changnan Wang, Xin Ni

**Affiliations:** a National Clinical Research Center for Geriatric Disorders, Central South University Xiangya Hospital, Changsha, People’s Republic of China; b International Collaborative Research Center for Medical Metabolomics, Central South University Xiangya Hospital, Changsha, People’s Republic of China; c Department of Physiology, Navy Medical University, Shanghai, People’s Republic of China; d The Key Laboratory of Exercise and Health Sciences of Ministry of Education, School of Kinesiology, Shanghai University of Sport, Shanghai, People’s Republic of China

**Keywords:** Hydrogen sulfide, hypoxia, NLRP3, inflammasome, adrenal insufficiency

## Abstract

**Objective:** Adrenocortical responsiveness is critical for maintaining glucocorticoids production and homeostasis during stress. We sought to investigate adrenocortical responsiveness during hypoxia in mice and the mechanisms responsible for the regulation of adrenal responsiveness.

**Methods:** (1) Adult male WT mice were randomly divided into four groups: normoxia, hypoxia (24h), hypoxia (72h), hypoxia (72h) + GYY4137(hydrogen sulfide (H_2_S) donor, 133mmol/kg/day); (2) WT mice were randomly divided into four groups: sham, adrenalectomy (ADX), sham+hypoxia, ADX+hypoxia; (3) *Cse*^*-/-*^ mice were randomly divided into two groups: *Cse*^*-/-*^, *Cse*^*-/-*^ +GYY4137.

**Results:** The circulatory level of corticosteroid induced by ACTH stimulation was significantly reduced in the mice with hypoxia compared with control mice. The mortality rate induced by lipopolysaccharide (LPS) increased during hypoxia. Cystathionine-γ-lyase (CSE) expression was significantly reduced in adrenal glands during hypoxia. GYY4137 treatment significantly increased adrenal responsiveness and attenuated NLRP3 inflammasome activation in mice treated by hypoxia and *Cse*^-/-^ mice. Furthermore, The sulfhydrated level of PSMA7 in adrenal gland was decreased in the mice with hypoxia and *Cse*^-/-^ mice. PSMA7 was S-sulfhydrated at cysteine 70. Blockage of S-sulfhydration of PSMA7 increased NLRP3 expression in adrenocortical cells.

**Conclusion:** Reduced H_2_S production mediated hypo-adrenocortical responsiveness and NLRP3 inflammasome activation via PAMA7 S-sulfhydration during hypoxia.

## 1. Introduction

Glucocorticoids (GCs) play key roles in the host’s response to various stress stimuli. Generally, stressors strongly stimulate the release of adrenocorticotrophic hormone (ACTH) from the pituitary gland, which in turn stimulates the secretion of GCs from the adrenal cortex [1,2]. The increase of GCs plays a crucial role in maintaining homeostasis during stress responses by modulation of metabolism, cardiovascular function, and immune system [1–3]. In some severe pathological conditions, such as sepsis, reversible adrenal insufficiency frequently occurs as evidenced by low basal GC level or low GC responses to ACTH stimulation [4–6]. Of note, adrenal insufficiency is associated with impaired responsiveness to vasopressors and a high mortality rate in critically ill patients [6,7]. Hypoxia is a common insult encountered by living organisms. It is known that hypoxia has detrimental effects on various tissues and organs, which can result in different responses to other insults in the organisms [8,9]. However, whether adrenal responses are altered under hypoxia conditions remains to be investigated.

Hydrogen sulfide (H_2_S) is the third gas signaling molecule identified in recent years [10,11]. In mammals, the body uses L-cysteine as a substrate to produce H_2_S under the catalysis of two main synthases, cystathionine-β-synthase (CBS), and cystathionine-γ-lyase (CSE). Many studies have reported that H_2_S plays important role in many physiological and pathological processes, including vasorelaxation, cardio protection, and inflammatory responses [12–15]. Our previous studies have shown that H_2_S produced by CBS and CSE in the adrenal cortex is involved in GC synthesis [16]. More recently, we have shown that circulatory H_2_S level is reduced in mice during hypoxia condition [17].

Based on the above background, we sought to explore whether adrenocortical responsiveness occurs under hypoxia condition, and then investigated the roles of H_2_S in this process and elucidated the underlying mechanisms. We revealed that hypoxia resulted in hypo-adrenocortical responsiveness in mice, which was associated with NLRP3 inflammasome activation in the adrenal gland. Reduced H_2_S production led to reduced adrenocortical responsiveness and NLRP3 inflammasome activation. Mechanically, we demonstrated that H_2_S S-sulfhydrated PSMA7 at cysteine 70 and subsequently inhibited NLRP3 expression to prevent hypoxia-induced adrenal insufficiency.

## 2. Materials and methods

### 2.1. Animal procedures

Adult male C57BL/6 mice (wild type, WT), 8- to 10-week-old, were purchased from the Shanghai SLAC Laboratory Animal Co. (Shanghai, China). *Cse*^–/–^ mice on a C57BL/6J background were generated by Shanghai Biomodel Organism Co., Ltd. Male *Cse*^–/–^ mice were used in the present study [17]. All animals were housed with regular light–dark cycles (lights on at 7:00 am, lights off at 7:00 pm) under a controlled temperature (22 ± 2°C) and humidity (50 ± 10%) and were provided standard diet and water ad libitum. All protocols involving animal studies were reviewed and approved by the Animal Care and Use Committee of Navy Medical University as well as the Ethnic Committee of Central South University Xiangya Hospital.

There were three sets of experiments. In the first set of experiments, adult male WT mice were randomly divided into four groups; two groups of mice were under normobaric hypoxia for 24 and 72 h, respectively. One group of mice underwent normobaric hypoxia condition for 72 h with GYY4137 (133 μmol/kg/day) treatment. One group of mice was under normoxia condition as control. For hypoxia treatment, the mice were placed in a hypoxia box (10% O_2_ and 90% N_2_) with a standard diet and water ad libitum [17]. GYY4137 was purchased from Sigma-Aldrich and dissolved in saline. The dosage of GYY4137 was chosen based on literature and our preliminary study [16–18]. The animals then underwent ACTH stimulation test or were sacrificed for the collection of blood and adrenal glands.

In the second set, WT mice were randomly divided into four groups. Two groups of mice underwent adrenalectomy (ADX). Two groups of mice underwent sham operation as controls. One week later, all mice were used for testing the mortality rate in response to lipopolysaccharide (LPS) treatment. Among them, two groups including a control group and an ADX group were placed in a hypoxia box (10% O_2_ and 90% N_2_). The other two groups were under normoxia conditions. Purified LPS extracted from the membrane of Escherichia coli 0111:B4 (Sigma-Aldrich, St. Louis, MO, USA) was dissolved in sterile pyrogen-free saline and injected into the peritoneal cavity at a dose of 30 mg/kg.

In the third set of experiments, *Cse^–/–^* mice were randomly divided into two groups, one group of mice were administered with GYY4137 (133μmol/kg/day), and the other group of mice was treated with saline. The mice were then undergoing ACTH stimulation test or sacrificed for collection of blood and adrenal glands at the time point of 72 h.

### 2.2. ACTH stimulation tests

The plasma corticosterone response to exogenous ACTH (Sigma-Aldrich) was determined as described previously [16,18]. First, dexamethasone (Sigma-Aldrich) was reconstituted in saline and injected i.p. at a dose of 5 μg/g body weight at 6:00 pm the night before and at 08:00 am the morning of the procedure. Two hours later, mice were anesthetized with intraperitoneal ketamine (80 mg/kg) and xylazine (10 mg/kg). A femoral arterial catheter was placed using aseptic procedures and ACTH (30 μg/kg) was infused. Arterial samples of 50 μL were obtained immediately before and at 15, 60, and 120 min after ACTH administration. Each sample was replaced with an equal volume of saline-containing heparin (100 units/mL).

### 2.3. Measurement of corticosterone levels

Corticosterone levels in serum were measured using a commercial ELISA kit from Cayman Chemical according to the manufacturer’s instruction [16]. This kit was characterized by a broad working range of 11.5–10,000 pg/mL. The speciﬁcity was 100% for corticosterone, 1.01% for progesterone, 0.25% for aldosterone, 0.18% for cortisol, 0.18% for testosterone, and 0.02% for cortisone, DHEA, DHEA-S, and pregnenolone. Intra-assay variation ranged from 8.4% to 13.5% and inter-assay variation ranged from 3.5% to 7.5%.

### 2.4. Western blot analysis

The tissues were homogenized in cold T-Per lysis buffer (Pierce Biotechnology, Inc. USA). Approximately 30μg/lane of protein samples were separated by 10–15% SDS-PAGE gel and subsequently transferred to nitrocellulose membranes. After blockage in 5% skim milk powder in 0.1% Tris-buffered saline/Tween 20 (TBST) for 2 h and incubation with antibodies against NLPR3 (D4D8T, Cell Signaling Technology), caspase-1 (sc-392736, Santa Cruz Biotechnology), GSDMD (YT7991, Immunoway Biotechnology), PSMA7 (15219-1-AP, Proteintech Group) overnight at 4°C at a dilution of 1:500 or 1:1000. Then the membranes were incubated with a secondary horseradish peroxidase-conjugated antibody for 1 h at room temperature. Immunoreactive proteins were visualized using an enhanced chemiluminescence western blotting detection system (Santa Cruz Biotechnology). The chemiluminescence signal from the membranes was quantified by the GeneGenome HR scanner using GeneTools software (SynGene). To control sampling errors, the ratio of band intensity to β-actin was obtained to quantify the relative protein expression level.

### 2.5. Cell culture

The Y1 cell line obtained from Chinese Academy of Sciences was maintained in a DMEM medium containing 5% FBS at 37°C in 5% CO_2_–95% air. The cells were plated in 12-well plate at a density of 4.5 × 10^5^ cells/well and cultured in the above media at 37°C in 5% CO_2_–95% air. The cells were treated with iodoacetamide (IAA, Sigma-Aldrich) at a dose of 2 mM for 24 h, and then the cells were harvested. In some cases, Y1 cells were transfected with wild type (WT) and mutant PSMA7 expression vectors by using the Xfect plasmid transfection reagents (Takara) according to the manufacturer’s instructions, and the cells were harvested for determination of NLRP3 expression level 24 h after transfection.

WT and mutant PSMA7 expression vectors were generated by Sangon Biotech, Shanghai, China. Briefly, the full-length PSMA7 complementary DNA (NC_000068.8) was cloned into pcDNA3.1 (+). The PSMA7 mutants (Cys63 to Ser63: GCG to CAG; Cys70 to Ser70: GTA to CAA and Cys91 to Ser91: GCC to CAC) were generated by using the PSMA7 construct and site-directed mutagenesis, the mutation results were verified by DNA sequencing.

### 2.6. Determination of the proteins with S-sulfhydration

S-sulfhydrated proteins were examined by using two methods. Maleimide assay was performed to identify potential S-sulfhydrated proteins as described previously [17,18]. Briefly, the adrenal gland tissues were homogenized in cold T-Per lysis buffer, and then centrifuged at 12,000×*g* for 10 min. The supernatants were collected and then incubated with 2 mM of Alexa Fluor 680 conjugated C2 maleimide for 2 h at 4°C with occasional gentle mixing followed by incubation with or without DTT (1 mM) for 1h at 4°C. The samples were used for SDS-PAGE gel electrophoresis. The protein bands with S-sulfhydration were collected from the gel for analysis by LC-MS/MS to identify the potential proteins that might be S-sulfhydrated.

For determination of S-sulfhydration of PSMA7, biotin-switch assay was conducted as described previously [17,18]. Briefly, adrenal tissues were homogenized in HEN buffer which consists of 250 mM Hepes–NaOH (pH 7.7), 1 mM EDTA and 0.1 mM neocuproine supplemented with 100 μM deferoxamine. After centrifugation at 12,000×*g* for 30 min at 4°C, the supernatants were incubated with HEN buffer (2.5% SDS and 20 mM methyl methanethiosulfonate) at 50°C for 20 min with frequent vortex. Acetone was then added to remove methyl methanethiosulfonate and the mixture was allowed to be precipitated at –20°C for 20 min. After centrifugation, the proteins were resuspended in HEN buffer containing 1% SDS (i.e. HENS buffer). The suspension was added with biotin–HPDP in dimethyl sulfoxide without ascorbic acid and incubated for 3 h at 25°C. Biotinylated proteins were precipitated by streptavidin–agarose beads. The proteins were then washed with the HENS buffer and then subjected for western blot analysis using antibodies against PSMA7.

### 2.7. Statistical analysis

Data were expressed as means ± SEM. Normal distribution was assessed by Shapiro–Wilk test. Statistical significance was determined according to sample distribution and homogeneity of variance. Statistical significance in experiments comparing only two groups was determined by two-tailed Student’s *t* test. The significance of the difference in mean values among more than two groups was evaluated by one-way analysis of variance (ANOVA) followed by post hoc analysis using Student–Newman–Keuls test. The significance of the difference in values of ACTH stimulation tests was evaluated by ANOVA followed by post hoc, pairwise analysis using Bennett’s test. To determine statistical significance between survival curves, Kaplan–Meier test was used. All statistical analyses were done with SPSS 16.0 (SPSS Inc., Chicago, IL). A *P-*value of less than 0.05 was considered significant.

## 3. Result

### 3.1. Hypoxia reduces adrenocortical responsiveness to ACTH and results in downregulation of CSE expression in adrenal glands

**Figure 1.**
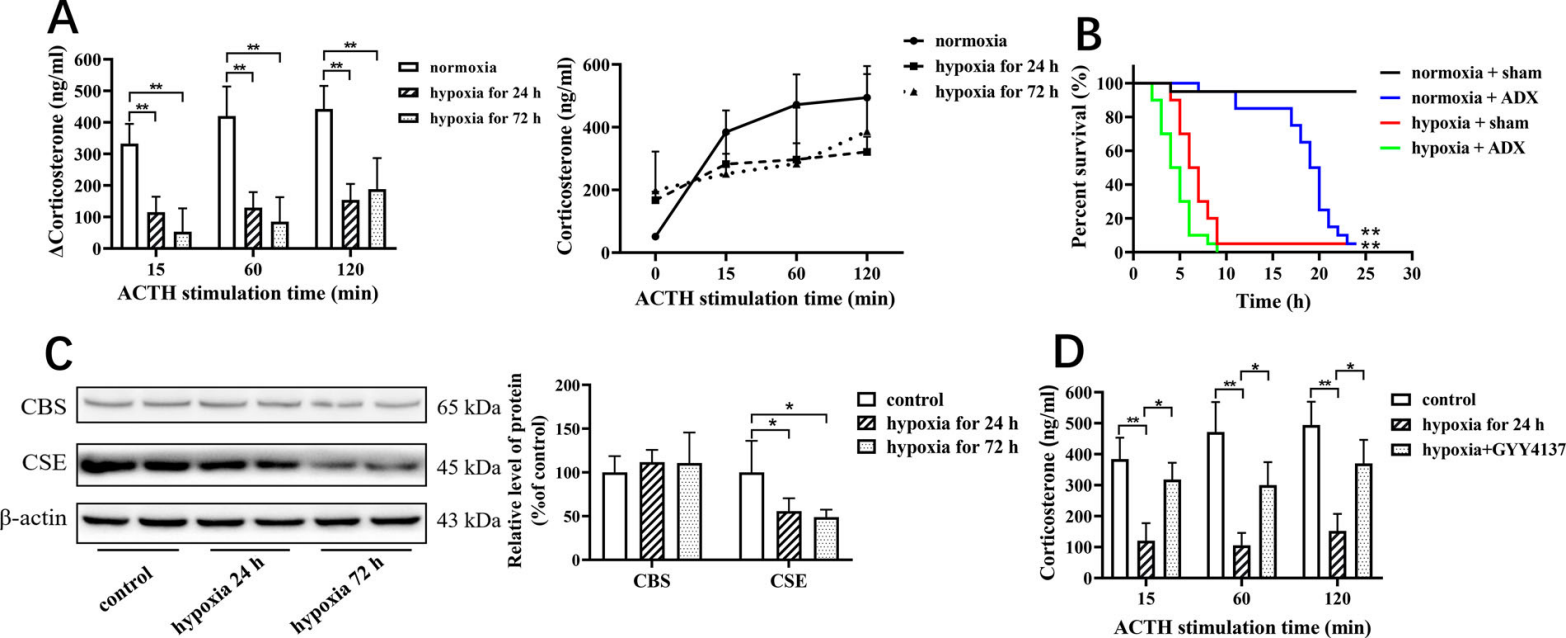
Hypoxia results in the downregulation of CSE expression in adrenal glands and reduces adrenocortical responsiveness to ACTH. (A) The mice underwent hypoxia for 24 and 72 h, respectively. ACTH stimulation test was applied to examine adrenocortical responsiveness. Left panel: Increase in corticosterone levels from baseline after ACTH stimulation at 15, 60 and 120 min. Right panel: The dynamic curve of ACTH stimulation test. Data were expressed as mean ± SEM. *n* = 8 in each group. **P < 0.01. (B) The mice underwent sham and ADX. One week later, all mice were administered with LPS at the dose of 30 mg/kg under normoxia and hypoxia. The mortality rate was determined. *n* = 10 in each group. **P < 0.01. (C) The mice underwent hypoxia for 24 and 72 h, respectively. The adrenal tissues were obtained for the determination of CBS and CSE protein expression using western blotting analysis. Left panel: Representative images of western blotting. Right panel: Statistical graph of western blotting. Data were expressed as mean ± SEM. *n* = 8 in each group. *P < 0.05. (D) The mice with hypoxia for 72 h were treated with GYY4137 at the dose of 133μmol/kg/day. ACTH stimulation test was applied to examine adrenocortical responsiveness. Data were expressed as mean ± SEM. *n* = 8 in each group. *P < 0.05, **P < 0.01.

As shown in Figure 1(A), the increased amount of corticosteroid in circulation induced by ACTH stimulation was significantly reduced in the mice with hypoxia for 24 and 72 h compared with control mice.

We then explored whether hypoxia affect the mice to counteract with stress insult. Mortality rate was estimated in the mice with LPS treatment. As shown Figure 1(B), under normoxia condition, 90% of the mice with sham operation were survive within 30 h after treatment, while all the mice with ADX were sacrificed within 30 h after treatment (*P* < 0.01 vs sham). Under hypoxia condition, 90% mice with sham were sacrificed within 10 h after treatment (*P* < 0.01 vs Sham with normoxia, *P* < 0.01 vs ADX with normoxia). There was no significant difference in mortality rate between sham and ADX group during hypoxia.

We also examined CSE and CBS expression in response to hypoxia insult. As shown in Figure 1(C), CSE level but not CBS level was significantly decreased in adrenal glands in mice in hypoxia compared with those in normoxia.

### 3.2. H_2_S donor GYY4137 treatment significantly alleviates hypo-adrenocortical responses under hypoxia

We then investigated the effects of H_2_S donor GYY4137 on hypo-adrenocortical responsiveness under hypoxia. As shown in Figure 1(D), GYY4137 treatment significantly increased corticosteroid level in response to ACTH stimulation.

### 3.3. Hypoxia causes NLRP3 inflammasome activation and pyroptosis in the adrenal gland and GYY4137 treatment reverses them

**Figure 2.**
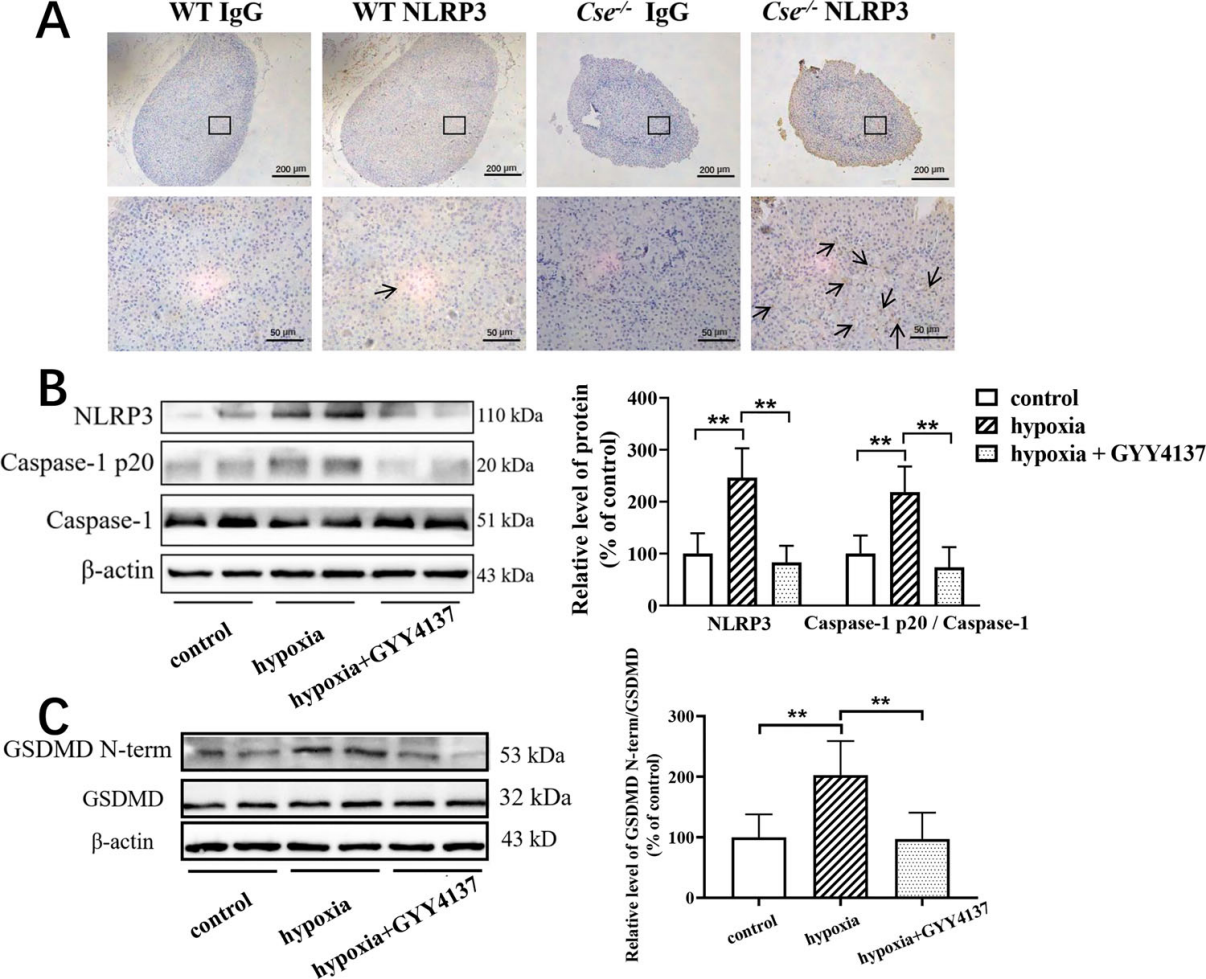
Hypoxia causes NLRP3 inflammasome activation and pyroptosis in adrenal gland and GYY4137 treatment reverses them. (A) NLRP3 protein expression in the adrenal gland of WT and *Cse^-/-^* mice was analyzed by immunocytochemistry. Representative images of NLRP3 in the adrenal gland of WT and *Cse^-/-^* mice. Arrows indicate NLRP3-positive staining. (B and C) WT mice with hypoxia for 72 h were treated with GYY4137 at the dose of 133 μmol/kg/day. The levels of NLRP3, Caspase-1 and cleaved caspase-1 p20 (B) and the levels of GSDMD and GSDMD N-terminal (C) in adrenal glands of the mice with or without GYY4137 treatment were determined by western blotting analysis. Data were expressed as mean ± SEM. *n* = 8 in each group. **P < 0.01.

**Figure 3.**
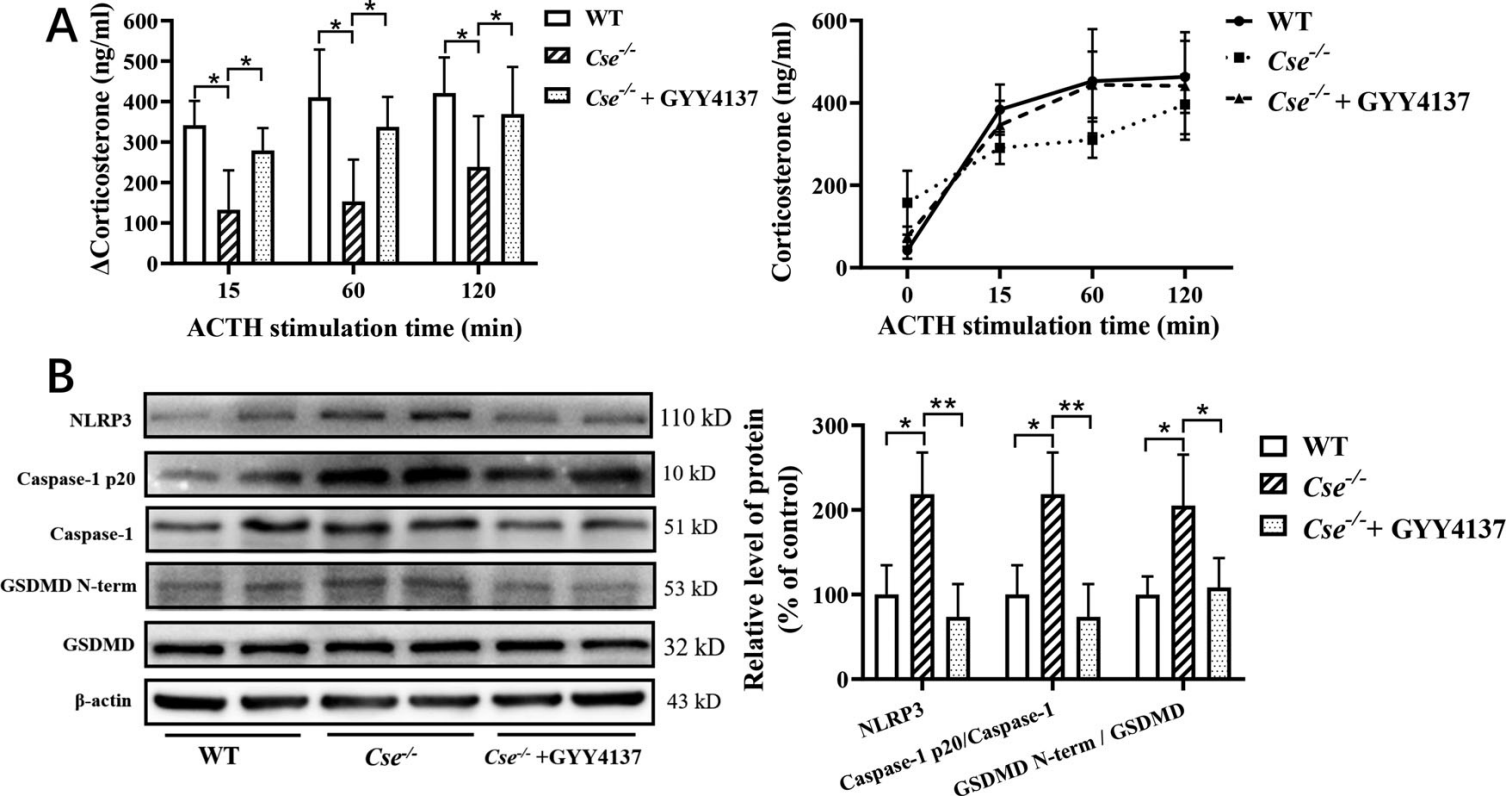
GYY4137 treatment reverses hyo-adrenocortical responsiveness, NLRP3 inflammasome activation and pyroptosis in adrenal glands in *Cse^-/-^* mice. *Cse^-/-^* mice were administered with GYY4137 at the dose of 133μmol/kg/day for 72 h. ACTH stimulation test was applied to examine adrenocortical responsiveness. The adrenal tissues were obtained for determination of CBS and CSE protein expression using western blotting analysis. (A) ACTH stimulation test. Left panel: Increase in corticosterone levels from baseline after ACTH stimulation at 15, 60 and 120 min. Right panel: The dynamic curve of ACTH stimulation test. (B) The protein expression of NLRP3, caspase-1, Caspase-1 p20, GSDMD and GSDMD N-terminal in adrenal glands. Left panel, Representative western blot analysis of NLRP3, Caspase-1, Caspase-1 p20, GSDMD and GSDMD N-terminal in adrenal glands. Right panel, The statistical graph of western blot results. Data were expressed as mean ± SEM. *n* = 8 in each group *p < 0.05, **P < 0.01.

Some studies have shown that H_2_S can regulate NLRP3 inflammasome activation in various tissues [19,20]. We, therefore, investigated NLRP3 inflammasome activation in adrenal glands of the mice under hypoxia. Firstly, we examined the distribution of NLRP3 expression in the adrenal gland. As shown in Figure 2(A), NLRP3 positive staining was distributed throughout the adrenal gland. Of note, a lot of positive staining was found in adrenal cortical cells. Western blotting analysis showed that NLRP3 expression level and cleaved caspase-1 level were significantly increased in the mice with hypoxia insult compared with the mice under normoxia (Figure 3B). GYY4137 treatment suppressed NLRP3 expression and cleaved caspase-1 level (Figure 2C).

It is known that NLRP3 inflammasome activation can lead to pyroptosis [21,22]. We, therefore, examined the GSDMD levels, and it was found that the level of GSDMD N-terminal fragment was significantly increased in the mice with hypoxia insult (Figure 2B), which was suppressed by GYY4137 treatment (Figure 2C).

### 3.4. CSE deficiency reduces adrenocortical responsiveness to ACTH and H_2_S donor treatment reverses it

To confirm down-regulation of CSE and decreased H_2_S level contribute to hypo-adrenocortical responsiveness, we applied ACTH stimulation test in *Cse*^–/–^ mice without and with GYY4137 treatment. As shown in Figure 3(A), *Cse*^–/–^ mice displayed reduced corticosterone responsiveness in ACTH stimulation. GYY4137 treatment significantly increased corticosteroid level in response to ACTH stimulation.

### 3.5. GYY4137 treatment reverses NLRP3 inflammasome activation and pyroptosis in adrenal glands of *Cse^–/–^* mice

As shown in Figure 3(B), *Cse^–^*^/–^ mice displayed increased NLRP3 expression level and cleaved caspase-1 level compared with wild type mice. The level of GSDMD N-terminal fragment was significantly increased in *Cse^–/–^* mice. GYY4137 treatment suppressed levels of NLRP3, cleaved caspase-1 and GSDMD N-terminal fragment in *Cse*^–/–^ mice.

### 3.6. Hypoxia and Cse deficiency result in a decrease in S-sulfhydration of PSMA7 level in the adrenal gland

**Figure 4.**
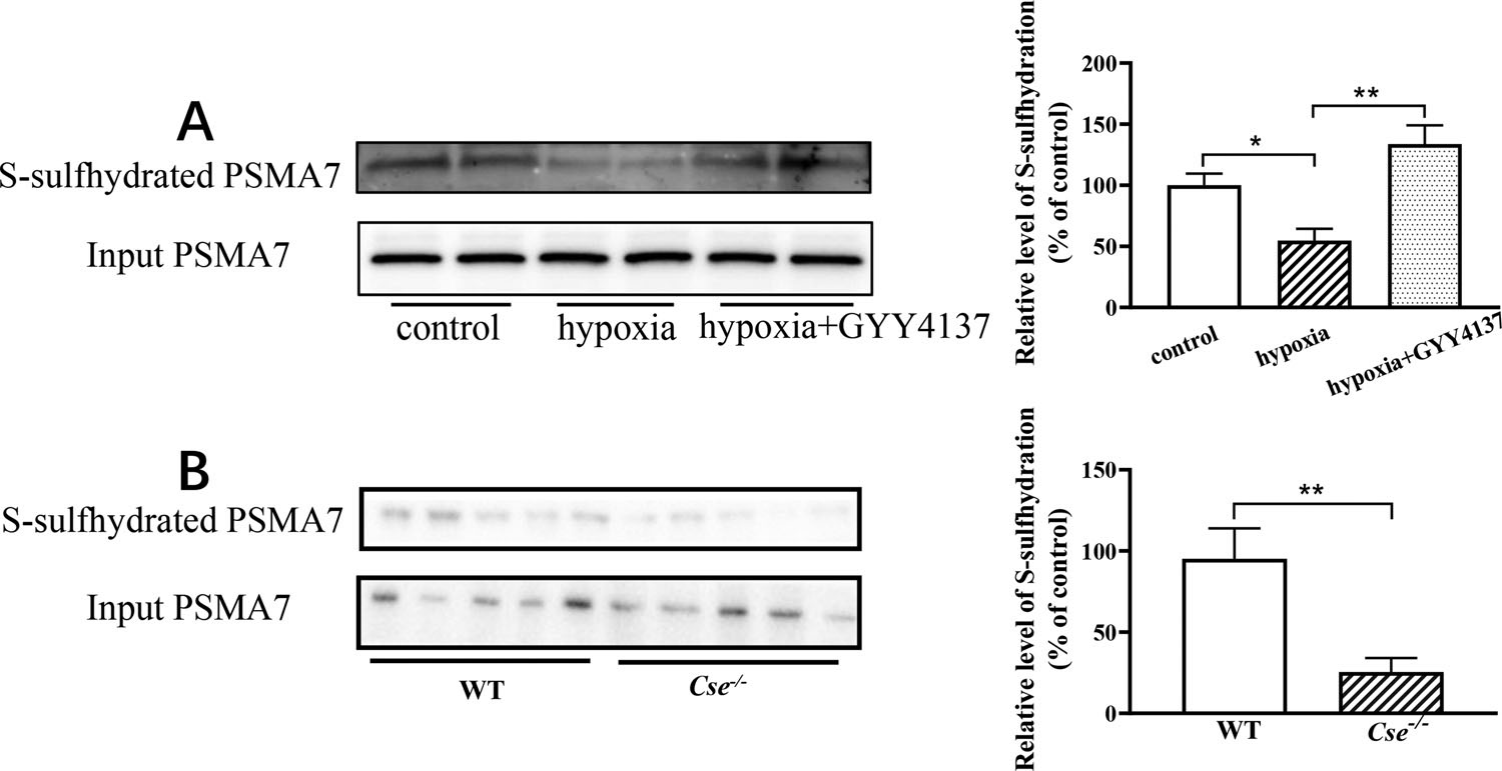
Hypoxia and CSE deficiency result in a decrease in S-sulfhydration of PSMA7 level in the adrenal gland. (A) WT mice with hypoxia for 72 h were treated with GYY4137 at the dose of 133μmol/kg/day. Biotin-switch assay was applied to determine the S-sulfhydration of PSMA7 level in adrenal glands. Left panel: The representative image of S-sulfhydration of PSMA7. Right panel: Statistical graph. (B) *Cse^-/-^* mice were administered with GYY4137 at the dose of 133μmol/kg/day for 72 h. A biotin-switch assay was applied to determine the S-sulfhydration of PSMA7 level in adrenal glands. Left panel: The representative image of S-sulfhydration of PSMA7. Right panel: Statistical graph. Data were expressed as mean ± SEM. *n* = 8. *P < 0.05, **P < 0.01.

Our previous study has shown that H_2_S can induce S-sulfhydration modification in some proteins of adrenal glands of mice [18]. Using a maleimide assay combined with mass spectrometry analysis, we found that there were more than 500 potential S-sulfhydrated proteins in mouse adrenal glands (Table 1). Proteasome subunit alpha type-7 (PSMA7) is a subunit of proteasome 20S core complex. It has been implicated that PSMA7 might be involved in NLRP3 inflammasome activation through MAVS degradation by ubiquitination [23,24]. We, therefore, investigated the level of PSMA7 S-sulfhydration in adrenal gland of the mice with hypoxia (Figure 4A). It was found that PSMA7 S-sulfhydration was significantly reduced in the adrenal glands of the mice with hypoxia insult. GYY4137 treatment could significantly increase PSMA7 S-sulfhydration level in the mice under hypoxia (Figure 4A).

**Table 1.** Mass spectrometry analysis of the S-sulfhydrated proteins.

Hlts	Protein mass (Da)	Sequence header
1	33,316	Glyoxalase domain-containing protein 4 [*Mus musculus*]
2	14,735	60S ribosomal protein [*Mus musculus*]
3	20,802	Ferritin light chain 1 [*Mus musculus*]
4	23,608	Glutathione S-transferase P [*Mus musculus*]
5	21,778	Peroxiredoxin-2 [*Mus musculus*]
6	34,127	NADH-cytochrome b5 reductase 3 [*Mus musculus*]
7	58,792	Glucosidase 2 subunit beta [*Mus musculus*]
8	27,854	Proteasome subunit alpha type-7 [*Mus musculus*]
9	15,142	Histone H2AX [*Mus musculus*]
10	16,837	Calmodulin-3 [*Mus musculus*]

We then confirmed that PSMA7 S-sulfhydration level was significantly decreased in *Cse^–/–^* mice (Figure 4B).

### 3.7. PSMA7 S-sulfhydration modulates NLRP3 expression in adrenocortical cells

**Figure 5.**
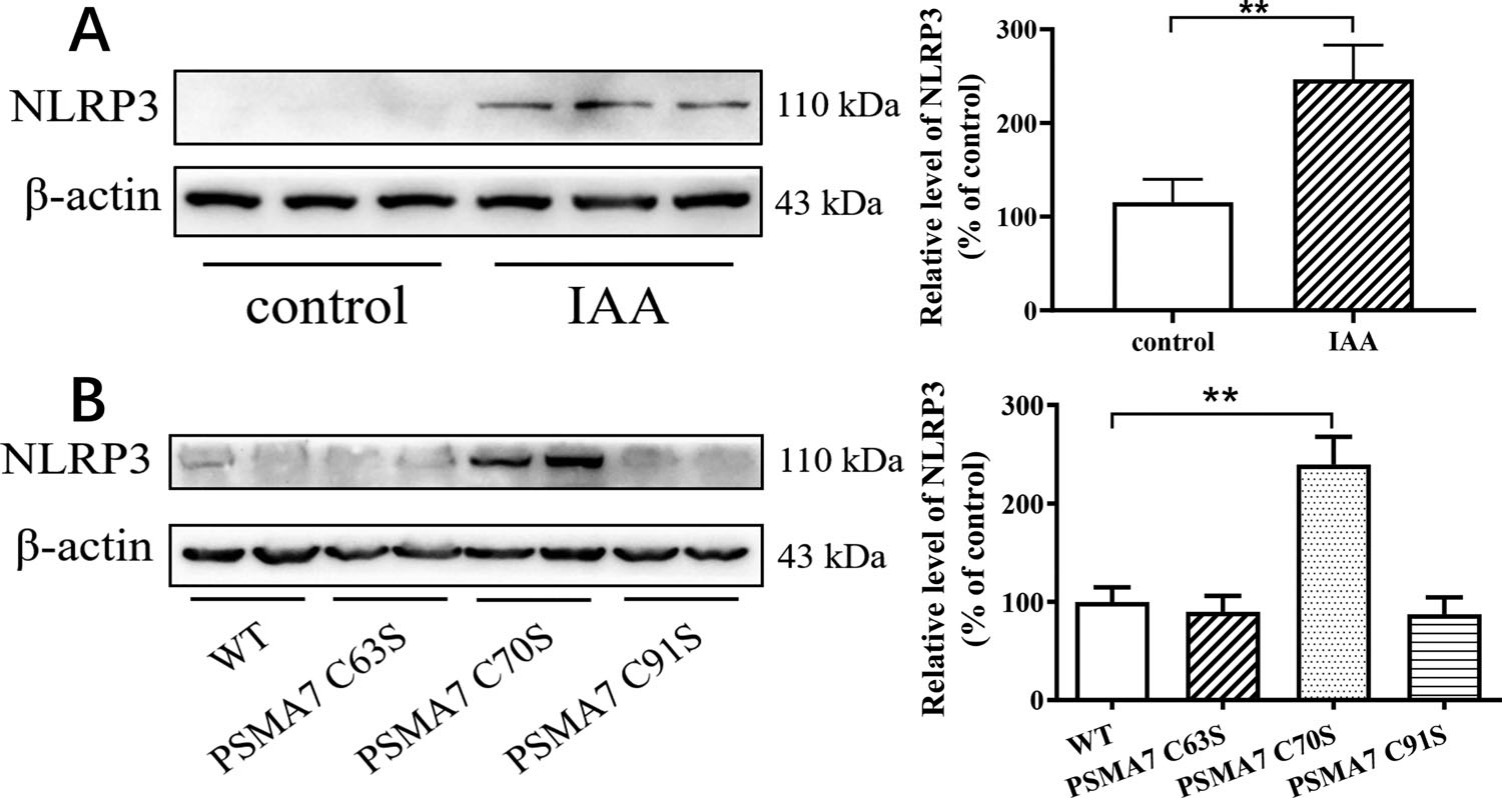
PSMA7 S-sulfhydration modulates NLRP3 expression in adrenocortical cells. (A) Y1 cells were treated with IAA (2 mM) or vehicle for 24 h and then harvested for the determination of NLRP3 protein expression. Left panel: The representative image of NLRP3. Right panel: Statistical graph. (B) Y1 cells were transfected with WT and various mutants of PSMA7. Left panel: The representative image of NLRP3. Right panel: Statistical graph. The cells were then harvested for the determination of NLRP3 protein expression. Data were expressed as mean ± SEM. *n* = 4 independent cultures. **P < 0.01.

As mentioned, PSMA7 is implicated to be involved in NLRP3 inflammasome activation, we then examined whether PSMA7 S-sulfhydration modulates NLRP3 expression using adrenocortical cell line Y1 cells. Iodoacetamide (IAA) can block S-sulfhydration modification of cysteine residue [17,25]. Figure 5(A) showed that treatment of Y1 cells with IAA for 24 h increased NLRP3 expression.

Next, we constructed mutant PSMA7 (Cys63 to Ser63, Cys70 to Ser70 and Cys91 to Ser91) expression vectors, i.e. PSMA7-C63S, PSMA7-C70S and PSMA7-C91S. All the mutant vectors confirmed by DNA sequencing (Figure S1A–C) were transfected into Y1 cells. As shown in the Figure 5(B), NLRP3 level was significantly increased in Y1 cells with PSMA7-C70S overexpression but not in cells transfected with the other two mutant vectors, suggesting that S-sulfhydration of PSMA7 at Cys 70 was involved in the regulation of NLRP3 protein expression.

## 4. Discussion

The present study has demonstrated that hypoxia could lead to hypo-adrenocortical responsiveness and increase the mortality rate in response to LPS insult. Further, we showed that H_2_S attenuated hypoxia-induced adrenal insufficiency, which was associated with NLRP3 inflammasome activation and pyropotosis in adrenal gland.

It is well known that hypoxia can lead to detrimental outcome in response to stress insults in humans [26,27]. Our findings that the LPS-induced mortality rate was significantly increased during hypoxia provided direct experimental evidence. GCs are the key hormones for the organisms in response to stress insults [3,4]. In critical care unit, patients with hypo-adrenocortical responsiveness have higher mortality rate [6,7]. Using an animal model, we previously demonstrated that LPS insult-induced mortality rate is significantly increased in the mice with adrenal insufficiency. In the present study, we showed that an increased mortality rate caused by LPS insult during hypoxia was associated with hypo-adrenocortical responsiveness.

H_2_S has mainly generated through CBS and CSE signaling pathways. We have previously shown that circulatory H_2_S level and CSE expression in many tissues are significantly decreased in response to hypoxia insult [17]. In the present study, we found that CSE expression was downregulated in adrenal glands during hypoxia. H_2_S supplement could significantly increase adrenocortical responsiveness during hypoxia. Moreover, adrenocortical responsiveness was significantly reduced in *Cse* deficiency mice, which was reversed by H_2_S treatment. Altogether, it suggests that H_2_S produced by CSE play a key role in maintaining GC production in response to ACTH stimulation. We have previously demonstrated that *Cbs* deficiency mice exhibit decreased corticosterone responsiveness to ACTH stimulation, which can be reversed by H_2_S treatment [18]. Thus it indicates that H_2_S is a key mediator in CBS and CSE modulation of adrenal cortex function. Of note, hypoxia insult suppresses CSE expression but not CBS expression in adrenal glands, while LPS treatment results in a decrease in both of CBS and CSE expression in adrenal glands [16], which suggests that CSE and CBS expression is susceptible to detrimental insults such as hypoxia and infection. Nevertheless, downregulation of CBS and CSE expression contributes to adrenal insufficient.

H_2_S directly interacts with the sulfhydryl groups of selective proteins yielding a hydropersulfide moiety (-SSH) in a process called S-sulfhydration. The physiological importance of S-sulfhydration is indicated by the large proportion of S-sulfhydrated proteins, such as c-jun, K_ATP_ and so on [18,20,28–30]. In the adrenal glands, we found that there were more than 500 potential sulfhydrated proteins. We previously identified S-sulfhydration of ATP5A1 in adrenal glands and have demonstrated that ATP5A1 is critical for the maintenance of ATPase activity, cell survival and steroidogenesis in adrenocortical cells [18]. In the present study, we found that S-sulfhydration of PSMA7 occurred under basal condition, and it was downregulated upon to hypoxia insult. PSMA7 is an α subunit of 20S proteasome core complex, and participates in protein degradation through ubiquitination-proteasome pathway [31]. Some studies indicate that NLRP3 inflammasome activation is controlled by PSMA7 because PSMA7 can interact with MAVEN, the latter recruits NLRP3 protein, thereby leading to NLRP3 inflammasome activation [23,24]. The present study showed that NLRP3 protein expression was regulated by S-sulfhydration of PSMA7 in adrenocortical cells. Moreover, we revealed that S-sufhydration of PSMA7 at cysteine 70 played a key role in the regulation of NLRP3 expression.

It is known that hypoxia can cause sterile inflammation in the tissues [32,33]. In recent years, NLRP3 inflammasome has been paid much attention since it is involved in many pathological processes [34]. Many studies have demonstrated that hypoxia insult can lead to NLRP3 inflammation activation in various tissues [35,36]. More recently, NLRP3 inflammasome activation is linked to pyroptosis in various cells as cleaved caspase-1 can cleave GSDMD [35,37,38]. Interestingly, some studies have demonstrated that NLRP3 inflammasome-dependent pyroptosis is involved in hypoxia-induced pathological changes in various tissues [35]. In consistence with the above studies, we found that pyroptosis occurred in adrenal glands in the mice upon to hypoxia insult and the mice with CSE deficiency, which was reversed by H_2_S treatment. We previously showed that CBS deficiency leads to apoptosis in the adrenal gland, which is prevented by H_2_S treatment.

## 5. Conclusion

**Figure 6.**
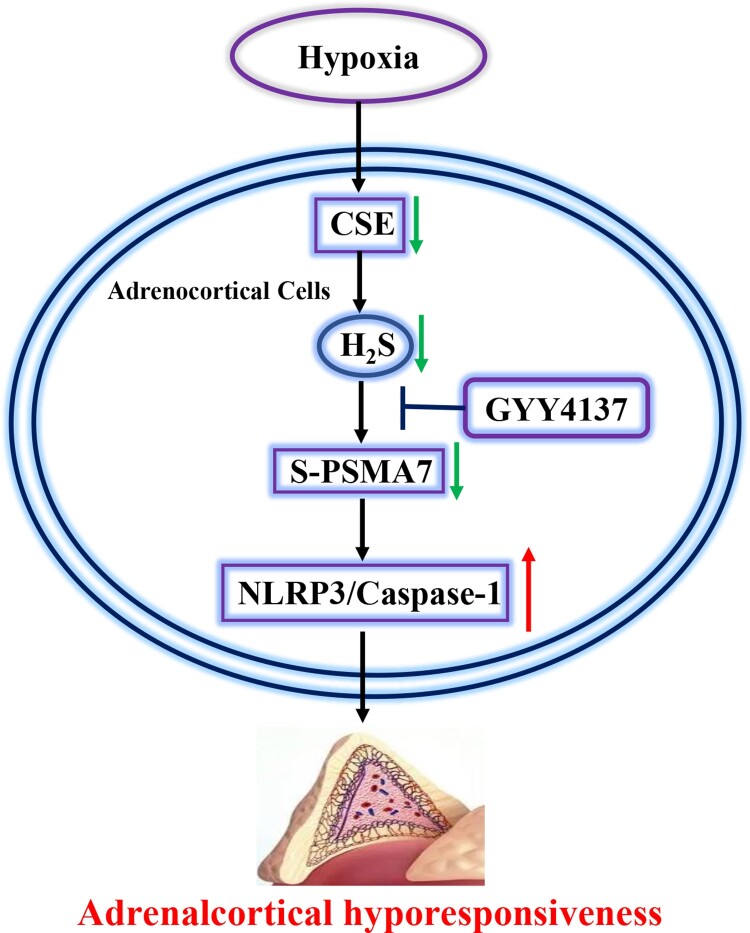
Working model: Hypoxia causes downregulation of CSE expression and reduced H_2_S level, which leads to reduced S-sulfhydration of PSMA7 at Cys70. Reduced S-sulfhydration of PSMA7 results in NLRP3 inflammasome activation in adrenal glands, thereby leading to adrenocortical insufficiency.

This study demonstrated that hypoxia causes adrenocortical insufficient, which is associated with NLRP3 inflammasome activation and pyroptosis in the adrenal gland. Reduced H_2_S production leads to NLRP3 inflammasome activation during hypoxia. H_2_S leads to S-sufhydration of PSMA7 at cysteine 70, subsequently suppresses NLRP3 expression in adrenocortical cells (Figure 6). Our data indicate that H_2_S is protective factor against hypoxia insult by maintaining adrenocortical responsiveness.

## Data Availability

The data used to support the findings of this study are available from the corresponding author upon request.
